# Inherited platelet disorders: Insight from platelet genomics using next-generation sequencing

**DOI:** 10.1080/09537104.2016.1195492

**Published:** 2016-10-11

**Authors:** Annabel Maclachlan, Steve P. Watson, Neil V. Morgan

**Affiliations:** ^a^ Institute of Cardiovascular Sciences, College of Medical and Dental Sciences, University of Birmingham, Birmingham, B15 2TT, UK

**Keywords:** Platelet disorders, genetics, bleeding, next generation sequencing

## Abstract

Inherited platelet disorders (IPDs) are a heterogeneous group of disorders associated with normal or reduced platelet counts and bleeding diatheses of varying severities. The identification of the underlying cause of IPDs is clinically challenging due to the absence of a gold-standard platelet test, and is often based on a clinical presentation and normal values in other hematology assays. As a consequence, a DNA-based approach has a potentially important role in the investigation of these patients. Next-generation sequencing (NGS) technologies are allowing the rapid analysis of genes that have been previously implicated in IPDs or that are known to have a key role in platelet regulation, as well as novel genes that have not been previously implicated in platelet dysfunction. The potential limitations of NGS arise with the interpretation of the sheer volume of genetic information obtained from whole exome sequencing (WES) or whole genome sequencing (WGS) in order to identify function-disrupting variants. Following on from bioinformatic analysis, a number of candidate genetic variants usually remain, therefore adding to the difficulty of phenotype–genotype segregation verification. Linking genetic changes to an underlying bleeding disorder is an ongoing challenge and may not always be feasible due to the multifactorial nature of IPDs. Nevertheless, NGS will play a key role in our understanding of the mechanisms of platelet function and the genetics involved.

## Introduction

Platelet disorders are a large group of diseases with variable bleeding diatheses ranging from mild to severe. They can be characterized quantitatively (i.e. thrombocytopenia) or qualitatively (i.e. function disorders) and can be either inherited or acquired. The majority of individuals with inherited platelet disorders (IPDs) have a platelet count within the normal range of 150–400 x 10^9^/L [1,2]. IPDs are considered to be rare with a frequency of 1: 10 000 but are likely to be under-recognized due to the difficulty of diagnosis, particularly in milder cases [1,3].

Platelets are small anucleate fragments, discoid in morphology, which originate from megakaryocytes in the bone marrow [3]. Upon vessel injury, when subendothelial matrix proteins become exposed, platelets undergo a series of rapid functional responses to form a vascular plug to limit blood loss [2–4]. Recent advances using mutant mice and human genetics have contributed greatly to the understanding of cellular and molecular mechanisms of the pathogenesis of IPDs. This includes the identification of the underlying genetic defects in heritable disorders, which is closely associated with their phenotype [5,6].

In the UK there are several groups investigating the causes of bleeding in patients with clinically diagnosed IPDs all involving next-generation sequencing (NGS). Additionally, groups from the University of Pavia and the University of Trieste in Italy collaborate on research into inherited thrombocytopenias and their associated genetics by NGS [7,8]. This review will discuss bleeding and IPDs with respect to mutations found following genomic investigation using NGS and the future of clinical investigation into the cause of unresolved bleeding disorders.

### Platelet disorders and testing prior to the NGS era

There are many types of disorders that fall under the umbrella term of IPDs. Most of the major IPDs associated with a strong phenotype are well characterized and were originally found by traditional methods of platelet function testing prior to our now “genomic era”. Examples of these include: Bernard–Soulier syndrome, Glanzmann thrombasthenia, Chediak–Higashi syndrome, Hermansky–Pudlak syndrome and Wiskott–Aldrich syndrome [4,9–12]. The classical gold standard platelet function assay – light transmission aggregometry (LTA), first described by Born – is still the most widely used test for the identification and diagnosis of IPDs as the responses to multiple concentrations of different agonists can be monitored [13]. Multiple electrode aggregometry (MEA) is also often used to measure whole blood impedance aggregation along with flow cytometry to measure the levels of platelet activation by markers such as glycoproteins and α-granule marker, P-selectin [14–17]. However, when platelet disorders are discovered as a result of the testing methods above, the outcomes usually stop short of giving the physicians and patients a final and definitive diagnosis. This explains the need to involve a level of genetic analysis either to confirm the observed phenotype or to suggest candidate variants when a diagnosis is not obvious.

### Genetic and genomics of platelet disorders

Genomics is defined as the study of the genetic material of an organism and its associated functions [18]. Genomics therefore aims to address all genes and their interrelationships in order to study and identify their combined influence on growth and development; this is in comparison to genetics, which mainly scrutinizes the function of a single gene [18]. The Human Genome project (HGP) began in the mid-1980s and was an international, collaborative project with the objective of completely mapping and improving the understanding of an individual’s entire nucleic material known collectively as our genome [19,20]. The initial sequence data was first published in the journal *Nature* in February 2001 when it was 90% complete and was finally proclaimed 99% complete in 2003 [21,22]. This project required and will continue to require new technologies to refine the draft and carry on research with regard to the human genome. Following the HGP, the 1000 Genomes Project was the first of its kind to sequence the genomes of a large group of individuals in order to provide a valuable resource to the scientific community on human genetic variation. The primary goal was to elucidate the genetic variants that have frequencies of at least 1% in the populations studied. The main drawback of the 1000 Genomes Project is that the samples are anonymous and therefore no phenotype data is available. However, the 1000 Genomes data can be combined with genome-wide association studies (GWAS) for additional variants beyond those already genotyped and allow for disease-associated genes, regions and variants to be researched in more depth. GWAS focus on the associations between single nucleotide polymorphisms (SNPs) and the varying phenotypes of a specific syndrome or disease typically seen in IPDs [23]. Family-based designs for GWAS are particularly beneficial as they enrich for genetic defects and have more power than unrelated individuals to detect genetic defects [24]. Family-based designs often include parent–offspring trios, sibling pairs, consanguineous families and large extended families with multiple affected family members [24]. The statistical principals underlying these designs such as the McNemar-type statistic are often complicated and will not be described here in detail [25–27].

Platelet genomics is a challenging area of research due to the absence of a nucleus. Platelets do have residual levels of megakaryocyte-derived RNA, and have been shown to make low amounts of proteins, most notably interleukin 1 [28–30]. The platelet genome is yet to be fully defined, but data enlightening on how genetic variation, at the level of gene transcription and translation, perturbs platelet function has been highly beneficial [28]. This has led to further analysis in recent years with the birth of more specialized techniques within genomics and more manageable costs of genomic investigation.

### Next-generation sequencing

DNA sequencing has been revolutionizing medical research since the invention of Sanger sequencing by Sanger *et al*. and the polymerase chain reaction (PCR) by Mullis et al. [31,32]. Traditional methods are now being replaced with NGS technologies and have led to an exponential reduction in the costs involved in sequencing whole genomes/exomes [6]. Whole genome sequencing (WGS) and whole exome sequencing (WES) are used to identify genetic factors causing human diseases, some of which were previously undetectable. These high-throughput experimental approaches are now more widely available than ever and have been adopted in both research and clinical settings in relation to patients with platelet and bleeding disorders [33]. It is well known that approximately 1.5% of the human genome contains protein-coding sequences (exons), within which 85% of alleles that underlie Mendelian disorders, that disrupt protein-coding sequences, reside [6]. Therefore, exome sequencing alone is enough to harbor enough data to uncover most rare and potential genetic disorders and the predisposing variants [6,34]. The first report of selectively sequencing all exomes successfully was published by Ng *et al*. in 2009 [35]. The method of WES can be read elsewhere and although there are several slightly different methods, they all follow the same principle for the rapid identification of protein-coding variants; mutations include missense and nonsense to small insertion/deletion mutations and splice site mutations [33,36]. WES can also be used to identify the genetic causes of multifactorial disorders that can be more common in bleeding disorders. There are some fundamental limitations of WES, the most obvious being that WES does not assess the impact of non-coding regions since it is limited to coding and splice site variants only [6]. The method is still time consuming and the coverage of regions of interest is not always complete. This has improved since the first experiments, where 8% of regions of interest were not captured by the enrichment strategy, but it is not expected that 100% coverage will ever be reached [33,35]. WES often remains not as cost effective as even a large targeted panel and still has difficulty in identifying repeat mutations and copy number variants, often leading to false positives [33]. Confirmation and validation of possible disease-causing variants by Sanger sequencing are nearly always required following WES as it has a very high level of coverage and the possibility of false-positive calls is little to none.

### The advance of genomic technologies in discovering genetic variants in IPDs

One of the main applications and challenges of NGS is proving that a certain genetic variant or variants are responsible for the clinical presentation of patients. In the case of bleeding and IPDs, the phenotypes can vary greatly from one individual to another even with the same genotype. This makes it very difficult to generate genotype–phenotype correlations and elucidate a disease-causing mechanism within these individuals. The first use of NGS in IPDs identified *NBEAL2* as the causative gene for grey platelet syndrome (GPS) [37]. Many causative mutations within the gene that encodes a BEACH domain protein cause differential bleeding severities in individuals with GPS [38]. In the past few years however, several research groups and their collaborators have employed NGS to discover the causes of unexplained bleeding; an example of a select few genes found using WES and/or WGS can be seen in Table I.

**Table I.** Novel genomic variants reported in genes recently discovered in patients with an inherited bleeding disorder following next-generation sequencing. Gene and phenotypes associated with variants are shown. Heterozygous nucleotide changes (unless indicated) present in patients with inherited bleeding and their predicted effects on the resulting RNA or protein are also shown. Genomic variations are numbered according to positions in the publication of the reference indicated. dbSNP ID is given for each variant if known or is a novel variant not reported in the available databases.

Gene Associated phenotypes	Genomic variation	Protein effect	Variation type	dbSNP ID	Reference
***NBEAL2***	c.256 A>G	p.Ile86Val	Missense	rs754407148	[37]
Thrombocytopenia, large platelets, lack of α-granules.	c.1163T>C	p.Leu388Pro	Missense	rs387907113	[37]
	c.1928 A>T	p.Glu643Val	Missense	rs387907114	[37]
	c.2044 >T	p.Ile682Phe	Missense	rs773164015	[37]
	c.6299 C>T	p.Pro2100Leu	Missense	rs387907115	[37]
	c.6802-2 A>AGGAGT		Splice site		[37]
	c.6806 C>T	p.Ser2269Leu	Missense	rs749896920	[12]
	c.7658 G>A	p.Gly2553Glu	Missense	rs144664865	[37]
***ACTN1***	c.64 G>A	p.Asp22Asn	Missense	rs387907346	[41]
Thrombocytopenia, large platelets	c.94 C>A	p.Gln32Lys	Missense	rs747559032	[40]
.	c.136 C>T	p.Arg46Trp	Missense	rs387907348	[41]
	c.137 G>A	p.Arg46Gln	Missense	rs387907345	[40,41]
	c.313 G>A	p.Val105Ile	Missense	rs387907350	[40]
	c.673 G>A	p.Glu225Lys	Missense		[40,41]
	c.751 G>C	p.Gly251Arg	Missense		[41]
	c.2210 C>A	p.Thr737Asn	Missense	rs387907349	[41]
	c.2212 C>T	p.Arg738Trp	Missense	rs387907347	[40,41]
	c.2255 G>A	p.Arg752Gln	Missense		[40,41]
	c.2289 G>A	p.Gly764Ser	Missense		[41]
	c.2305 G>A	p.Glu769Lys	Missense		[41]
***SRC***	c.1579 G>A	p.Glu527Lys	Missense		[42]
Thrombocytopenia, sometimes large platelets, lack of α-granules.					
***SLFN14***	c.652 A>G	p.Lys218Glu	Missense		[45]
Thrombocytopenia, reduced δ-granules.	c.657 T>A	p.Lys219Asn	Missense		[45]
	c.659 T>A	p.Val220Asp	Missense		[45]
	c.667 C>T	p.Arg223Trp	Missense	rs766076920	[7]
***ETV6***	c.641 C>T	p.Pro214Leu	Missense	rs724159947	[49]
Thrombocytopenia, hematologic malignancy.	c.1106 G>A	p.Arg369Gln	Missense	rs724159946	[49]
	c.1195 C>T	p.Arg399Cys	Missense	rs724159945	[49]
***FYB***	c.393 G>A^Hom^	p.Trp131Stop^Hom^	Nonsense		[51]
Thrombocytopenia, small platelets.					
***PRKACG***	c.222 C>G^Hom^	p.Ile74Met^Hom^	Missense	rs724159972	[52]
Thrombocytopenia, giant platelets.					
***RBM8A***	c.-21 G>A		Regulatory SNP	rs139428292	[53]
Thrombocytopenia, absence of radius bones.	c.67+32G>C		Regulatory SNP	rs201779890	[53]

The BRIDGE consortium was introduced as the NIHR BioResource funded body for projects involving NGS related to rare disease-causing variants. The BRIDGE-Bleeding and Platelet Disorders (BDPs) study (B20) takes an alternative approach to the discovery of novel and known variants by high throughput sequencing (HTS) of a large patient cohort (>1000 donors) and annotation with adapted Human Phenotype Ontology (HPO) terms (https://bridgestudy.medschl.cam.ac.uk/bpd.shtml) [39]. Their aim is to characterize cases with similar HPO terms and variants in the same gene to aid in gene discovery [39]. The BRIDGE-BPD study used their novel approach to gene discovery using HPO-encoded phenotype data for novel variants in *ACTN1* along with other genes that have been implicated with bleeding including *MYH9, ACTN1* being originally reported by Kunishima *et al*. in 2013 [39,40]. *ACTN1* mutations cause congenital macrothrombocytopenia as they affect the α-actinin protein involved in the organization of the platelet cytoskeleton [40]. The platelet macrocytosis indicates that the latest phases of megakaryopoiesis are affected [41]. A gain-of-function mutation in *SRC* was also found to cause bleeding and other bone pathologies following WGS and HPO patient coding within the BRIDGE-BDP study [42]. In order to ensure accurate annotation of the particular phenotypes, the study added 80 terms and associated symptoms to HPO in parallel with the development of the software hpoPlot that summarizes the HPO codes of the cases [43]. It was hypothesized that individuals with rare coding variants in the disease gene would cluster on the basis of the HPO-encoded phenotypes they were given upon recruitment to the study [39]. The data that was generated suggested that despite minor anomalies, bleeding disorders caused by common genetic backgrounds would tend to cluster on the basis of their HPO terms [39]. This approach to gene discovery was pioneered in bleeding and platelet disorders, but is applicable to other rare diseases. The HPO data can be linked to large online databases such as OMIM, which can be used to prioritize disease gene candidates based on comparison to other ontological phenotype terms from the scientific literature [39].

The Genotyping and Phenotyping of Platelets (GAPP) study run from the University of Birmingham has recruited over 700 participants with bleeding disorders of unknown cause from over 25 collaborating Haemophilia Comprehensive Care Centres across the UK and involves collaborations with the Universities of Bristol, Nottingham and Sheffield (http://www.birmingham.ac.uk/research/activity/cardiovascular-sciences/research/platelet-group/platelet-gapp/index.aspx) [1]. The GAPP study team uses a combination of platelet phenotyping and WES in a different approach to variant discovery within platelet genomics with the aim of identifying the likely causative gene(s) within each recruited individual [1,44]. They emphasize the importance of the genotype–phenotype correlation to improve the classification and diagnosis of patients with novel or known platelet and bleeding disorders [1]. However, since the phenotype can be the result of multiple alleles with different pathogenic effects, interpreting genetic variants remains difficult. Patients with novel *SLFN14* variants as well as those with *RUNX1* and *FLI1* variants were implicated in bleeding and discovered through the GAPP study methodology, which shows effective proof of principle [45,46]. The *SLFN14* variants were narrowed down from eight potential disease-causing single nucleotide variants (SNVs) shared by the three patients of the same family in the initial investigation. The affected individuals present with thrombocytopenia and excessive bleeding disproportionate to their platelet counts [45]. The platelets also have a reduced number of δ-granules and therefore ADP secretion is also reduced [45]. The GAPP study continues to recruit patients from their collaborating hemophilia centers in the UK and beyond.

The ThromboGenomics platform has also been implementing NGS with regard to bleeding, thrombotic and platelet disorders (http://www.thrombogenomics.org.uk). They have developed a targeted gene capture platform that encompasses approximately 70 candidate genes with the aim of providing a multi-gene high-throughput sequencing platform to obtain diagnoses for patients with suspected bleeding or platelet disorders [47]. Again the use of the HPO term-based coding of patient phenotypes is being utilized in order to elucidate genotype–phenotype correlations [47]. The ThromboGenomics platform has used WGS data and is critically dependent on the recruitment of patients with strong, clinically well-characterized phenotypes. WES has been carried out on the genes *ITGA2B* and *ITGB3* with input from ThromboGenomics in order to identify novel variants within these genes and predict whether the said variants are likely to cause Glanzmann Thrombasthenia [48]. ThromboGenomics has multiple national and international collaborators that are involved at various levels to continue the development of the platform.

Other genes implicated in IPDs have been discovered outside of the main research groups by NGS technologies. Variants within the gene *ETV6* have recently been shown to cause thrombocytopenia with susceptibility to hematologic malignancy [49]. WES of the affected family members in the study group was carried out to discover five potential protein altering variants [49]. However, Sanger sequencing confirmed the heterozygous germline ETV6 variant as the definite cause of their bleeding phenotype [49]. The known pathogenic variant of *FYB* also causes thrombocytopenia along with a reduced percentage of mature platelets in the bone marrow [50,51]. This was discovered following WES, homozygosity mapping and extensive Sanger sequencing and genotyping where the segregation of the variant was confirmed [50]. A mutation in the *PRKACG* gene has been identified to cause a new form of macrothrombocytopenia as it leads to a defect in proplatelet formation [52]. Again this discovery was enabled by WES of the proband, another affected individual and other healthy family members [52]. As in most cases, other variants were found through bioinformatic analysis, but as *PRKACG* is highly conserved and implicated in platelet processes this remained their best candidate [52]. Mutations in the regulatory regions of the gene *RBM8A* are now known to cause thrombocytopenia with absent radii (TAR) syndrome, again discovered by WES [37,53]. These advances illustrate the importance and recent increase in the popularity of using NGS to discover genetic variants in IPDs.

### Challenges and opportunities

One of the main challenges arises with the development of bioinformatic analysis tools that are already lagging behind the advances in the substantial amount of data generated by NGS. This often leads to the analysis of bioinformatic data post NGS being the rate-limiting step in the process of gene identification. Therefore, the second challenge faced within platelet genomics is the identification of the causal gene and variant among multiple possible variants and link it to disease and function [34]. This is often equivocal and several candidates remain. Presently, sequence variant analysis is imperfect and a tremendous amount of work is needed to introduce a large-scale statistical automated framework for the calling of variants [54,55]. There have been standards and guidelines published for the interpretation of sequence variants as recommended from the American College of Medical Genetics and Genomics (ACMG) and the Association for Molecular Pathology [54]. The report recommends the use of specific consensus terminology – ‘pathogenic’, ‘likely pathogenic’, ‘uncertain significance’, ‘likely benign’ and ‘benign’ to describe variants identified in possible disease-causing genes based on evidence for a set of criteria and the strength of the criteria [54]. Another potential challenge arises with the inevitable discovery of genetic variants not related to the disease state in question, the so-called incidental or secondary findings [56]. This is a complex issue with many confounding factors involving ethics. The issue faced by clinicians is, should they disclose secondary findings with potential clinical relevance to their patients and if so, when and how? The interpretation of the information delivered would require education and in relation to some inherited bleeding disorders possible genetic counseling. The ACMG has published a policy statement concerning NGS with emphasis on incidental findings in clinical pretesting, genetic testing and the reporting of results [57]. The recommendations of the ACMG Working Group on Incidental Findings in Clinical Exome and Genome Sequencing have also been published and outline a list conditions, genes and variants they recommend for return of incidental findings in clinical sequencing [58]. This is deemed by many to go beyond targeted sequencing and true incidental findings to suggest automatic screening for genetic conditions in all patients undergoing WES/WGS [59]. It is a controversial area of interest that requires further social and professional debate, which should both proceed with ethical caution and concern [59].

It is all very well being able to elucidate mutations from rare disease-causing variants; however, the view that there is little purpose in finding the genetic causes of diseases is held by some. This is because slow progress has been made improving the understanding of the consequences of the genetic variants we already know about [33]. What has been discovered to cause bleeding through NGS though suggests that this argument does not hold. We now have knowledge of platelet genes that are known or predicted to have a role in the regulation of platelets along with platelet physiological processes and we are therefore able to diagnose patients with specific IPDs as a result [5]. It is important to emphasize the need for cell biology to explore novel unclassified variants in order to investigate expression analysis and protein function to ensure a variant is well characterized and proven to be functionally disrupting [1]. As more data is accumulated about the genetics of bleeding and platelet disorders, the list of candidate variants will decrease. Therefore, the likelihood of finding a disease-causing mutation improves even within platelet defects where this will always remain a challenge due to their multifactorial nature [33].

## Conclusion

The advances that have been described impact the medical management of individuals suffering from IPDs and provide insights into the mechanisms of diseases [34]. Future directions following WES are to employ WGS to search for mutations in observed bleeding phenotype–genotype mismatches. WGS does have comprehensive genomic coverage but is extremely limited in its use by its high price, although this is now reducing, and heavy analytical burden. Once these issues have been addressed and the bioinformatics tools have been improved and made more user-friendly; along with databases improving with more and more variants being deposited into the said databases, it may become the sequencing technology of choice for many. However, a sustained collaboration among research scientists, clinicians and commercial vendors, for the machines and materials needed, is required for this next phase of NGS [60]. For the moment though, WES is highly successful and this robust genetic technique will remain at the forefront of platelet genomic research.
